# Supplementary material for: Linker histone H1.2 and H1.4 affect the neutrophil lineage determination
Source: eLife. 2020 May 11;9:e52563. doi: 10.7554/eLife.52563 (PMC7250579; doi:10.7554/eLife.52563)
Supplement: Supplementary file 2. — Table of reagents, cell lines, genetically modified organisms, others. [file elife-52563-supp2.docx]

**Template:**

| **Key Resources Table** | | | | |
| --- | --- | --- | --- | --- |
| **Reagent type (species) or resource** | **Designation** | **Source or reference** | **Identifiers** | **Additional information** |
| Strain, strain background (*E.coli*) | Alexa Fluor 488  conjugated *E.coli* | Thermo Fisher Scientific, E13231 |  | Used for phagocytosis assays |
| strain, strain background (*E.coli*) | Stbl3 chemically competent cells | Thermo Fisher Scientific, C737303 |  | Competent RecA negative bacteria used for all lentiviral plasmids |
| genetic reagent (*Mus Musculus*) | Histone H1.2/H1.4 deficient mouse | Gift from Arthur I Skoultchi, Fan et al. Mol Cell Biol, 23(2003), pp.4559-72 |  | Mice double-deficient for linker histones H1.2 and H1.4 |
| cell line (Homo Sapiens) | PLB-985 | Zhen et al. Proc Natl Acad Sci U S A, 90(1993), pp.9832-6 | RRID:CVCL_2162 |  |
| cell line (Homo Sapiens) | THP-1 | DSMZ-German Collection of Microorganisms and cell cultures, ACC16 | RRID:CVCL_0006 |  |
| cell line (Homo Sapiens) | HEK293T | ATCC, CRL-11268 |  | Used for generation of lentiviral particles |
| transfected construct (Homo Sapiens) | lentiCRISPRv2 | Addgene plasmid #52961 | RRID:Addgene_52961 | Plasmid backbone to deliver all sgRNAs in this paper . All sgRNA sequences are provided in supplementary file 1 |
| Transfected construct (Homo Sapiens) | lentiCRISPRv2-Blast | This paper |  | Same plasmid backbone as lentiCRISPRv2, exchange of Cas9 to Blasticidin resistance to generate double knockout lines |
| Transfected construct (Homo Sapiens) | lentiCRISPRv2 GECKO library | Addgene, Shalem et al. Science, 343(2014), pp.84-87 |  | Genome-wide sgRNA library |
| Antibody | H1.2 (rabbit anti-human, IgG polyclonal) | PA5-32009, Invitrogen | RRID:AB_2549482 | Lot no. RK2200901B, used for western blot (1:1000) |
| Antibody | H1.4 (rabbit anti-human, IgG, monoclonal) | 41328S, Cell Signaling | RRID:AB_2799199 | Lot no. 1, used for western blot (1:1000) |
| Antibody | MPO (rabbit anti-human, polyclonal) | A0398, Dako | RRID:AB_2335676 | Used for western blot (1:2500) |
| Antibody | Histone H3 (rabbit anti-human, monoclonal) | ab1791, Abcam | RRID:AB_302613 | Used for western blot (1:1000) |
| Antibody | GAPDH (rabbit anti-human, monoclonal) | GAPDH (14C10) Rabbit mAb #2118 | RRID:AB_10693448 | Used for western blot (1:2500) |
| Antibody | PE-Cd11b (mouse anti-human, monoclonal) | 557321, BD | RRID:AB_396636 | Lot no. 8334634 Used for flow cytometry (1:100) |
| Antibody | BV-421-Cd11b (Rat anti-mouse, monoclonal) | 562605, BD | RRID:AB_11152949 | Lot no. 5300879, used for flow cytometry (1:100) |
| Antibody | PE-Ly6G (Rat anti-mouse, monoclonal) | 551461, BD | RRID:AB_394208 | Lot no. 4246573, used for flow cytometry (1:100) |
| Antibody | APC-Cy7-SiglecF (Rat anti-mouse, monoclonal) | 565527, BD | RRID:AB_2732831 | Lot no. 7244817, used for flow cytometry (1:100) |
| Antibody | V500-Cd45 (Rat anti-mouse, monoclonal) | 561487, BD | RRID:AB_10697046 | Lot no. 8116601/7137971, used for flow cytometry (1:100) |
| Antibody | FITC-Cd3 (Rat anti-mouse, monoclonal) | 561798, BD | RRID:AB_10898341 | Lot no. 7222701, used for flow cytometry (1:100) |
| Antibody | PE-Cy7-Cd4 (Rat anti-mouse, monoclonal) | 552775, BD | RRID:AB_394461 | Lot no. 6214844, used for flow cytometry (1:100) |
| Antibody | APC-Cd8a (Rat anti-mouse, monoclonal) | 561093, BD | RRID:AB_10563416 | Lot no. 8099799, used for flow cytometry (1:100) |
| Antibody | PE-Cd115 (Rat anti-mouse, monoclonal | 565249, BD | RRID:AB_2739132 | Lot no. 6050996/6286744, used for flow cytometry (1:100) |
| Antibody | PerCP-Cy5.5 Gr1 (Rat anti-mouse, monoclonal) | 561103, BD | RRID:AB_10562568 | Lot no. 7076948, used for flow cytometry (1:100) |
| Antibody | APC-Siglec-8 (mouse anti-human, monoclonal) | BD 347105 | RRID:AB_2561401 | Used for flow cytometry (1:100) |
| Antibody | Galectin-10 (rabbit anti human, polyclonal) | Abcam, ab231964 |  | Used for western blot (1:500) and immunofluorescence (1:200) |
| recombinant DNA reagent | qRT-PCR primers | Oligonucleotides from Sigma |  | All primer sequences are described in supplementary file 1 |
| Recombinant DNA reagent | Primers for amplicon generation in CRISPR screen | Shalem et al. Science, 343(2014), pp.84-87 |  | PCRs done as described |
| commercial assay or kit | DNeasy blood and tissue kit | Qiagen |  | To isolate DNA from GECKO screen samples |
| Commercial assay or kit | RNeasy kit | Qiagen |  | To isolate total RNA for RNA-seq experiments |
| Commercial assay or kit | Zyppy plasmid mini kit | Zymo Research, D4036 |  | Plasmid minipreps |
| Commercial assay or kit | Zymoclean gel DNA recovery kit | Zymo Research, D4001/D4002 |  | Gel extractions, used for purification of DNA for PLB-985 ko clone sequencing |
| Commercial assay or kit | Phusion High-Fidelity DNA Polymerase | New England Biolabs, M0530 |  | For PCR amplification of genomic DNA sequences to sequence PLB-985 ko clones |
| Commercial assay or kit | cDNA synthesis kit | High Capacity cDNA Reverse Transcription Kit, 4368814, Thermo Fisher Scientific |  |  |
| Commercial assay or kit | SYBR green | 4385612, Thermo Fisher Scientific |  |  |
| Commercial assay or kit | CD14 monocyte isolation kit | 130-050-201, Miltenyi Biotec | RRID:AB_2665482 | Used for isolation of primary monocytes |
| Commercial assay or kit | Lineage negative selection kit | 130-110-470, Miltenyi Biotec |  | Used for isolation of lin- BM cells from mouse |
| Commercial assay or kit | Human eosinophil isolation kit | 130-092-010,  Miltenyi Biotech |  |  |
| Commercial assay or kit | Murine CXCL1 ELISA | DY453, R&D systems |  |  |
| Commercial assay or kit | Murine IL-17 ELISA | DY421, R&D systems |  |  |
| Commercial assay or kit | Murine G-CSF ELISA | MCS00, R&D systems |  |  |
| chemical compound, drug | Puromycin | Sigma, P9620 |  | For selection of Puromycin-resistant PLB-985 |
| Chemical compound, drug | Blasticidin | Invivogen, #ant-bl-1 |  | For selection of Blastidicin-resistant PLB-985 |
| Chemical compound, drug | Cytochalasin B | Sigma, C6762 |  | To block phagocytosis |
| Chemical compound, drug | Lipofectamine 2000 | Thermo Fisher Scientific, 11668019 |  | For transfection of HEK293T cells |
| Chemical compound | SYTOX Green nucleic acid stain | Thermo Fisher Scientific |  | Used in live/dead stains |
| Chemical compound | DAPI | 4′,6-diamidino-2-phenylindole, Sigma |  |  |
| Chemical compound | Luminol | 11050, AAT-Bioquest |  | Used to measure production of ROS |
| Chemical compound | HRP | 31941, Serva |  | Used to measure production of ROS |
| Chemical compound | PMA | P8139, Sigma |  | Used to stimulate neutrophils and PLB-985 cells |
| Chemical compound | A23187 | Santa Cruz Biotechnology Inc. |  | Calcium ionophore used to stimulate PLB-985 |
| Chemical compound | GATA-1 inhibitor | anagrelide, SML0846, Sigma |  |  |
| Chemical compound | GATA-2 inhibitor | K-7174, HY-12743A, MedChemExpress |  |  |
| Chemical compound | IL-3, IL-5, IL-9, GM-CSF, SCF, G-CSF | Peprotech |  | Recombinant murine cytokines used for differentiation of lineage negative BM cells and for incubation of bone marrow cells with G-CSF |
| Chemical compound | Histopaque-1119 | 1119, Sigma |  | Used for isolation of neutrophils and PBMCs from human blood |
| Chemical compound | Histopaque-1077 | 10771, Sigma |  | Used for enrichment of viable PLB-985 after differentiation |
| Chemical compound | Percoll | GE Healthcare |  | Used for isolation of neutrophils from human blood |
| Chemical compound | Trizol | 15596018 |  | For RNA isolation |
| Chemical compound | Fix/lyse solution | eBioscience 1 Step Fix/Lyse Solution (invitrogen, 00-5333-54) |  | For fixation of murine blood prior to flow cytometry |
| Chemical compound | Dimethylformamide | DMF, D4551, Sigma |  | Used for differentiation of PLB-985 |
| Other | Lenti-X GoStix Plus | Takara, 631281 |  | For quantification of lentivirus in supernatants of transfected HEK293T |
| other | RPM-1640 | Gibco |  | Propagation of PLB-985, HEK293T, THP1 |
| Other | RPMI-1640 w/o phenol red | Gibco |  | Assay medium |
| Other | Opti-MEM | Gibco |  | For transfection of HEK293T |
| Other | IMDM | Gibco |  | For differentiation of murine lineage negative stem cells in vitro |
| Other | L-Glutamine | 25030-024, Gibco |  | Media supplement |
| Other | Penicillin/Streptomycin | 15140-122, Gibco |  | Media supplement |
